# A Combination of Atorvastatin and Aspirin Enhances the Pro-Regenerative Interactions of Marrow Stromal Cells and Stroke-Derived Monocytes *In Vitro*

**DOI:** 10.3389/fphar.2021.589418

**Published:** 2021-04-20

**Authors:** Nikunj Satani, Xu Zhang, Kaavya Giridhar, Natalia Wewior, Chunyan Cai, Jaroslaw Aronowski, Sean I. Savitz

**Affiliations:** ^1^ Department of Neurology, Institute for Stroke and Cerebrovascular Disease, McGovern Medical School at UTHealth, Houston, TX, United States; ^2^ Center for Clinical and Translational Sciences, McGovern Medical School at UTHealth, Houston, TX, United States

**Keywords:** statins, aspirin, combination therapy, mesenchymal stromal cells, cell therapy, ischemic stroke

## Abstract

**Background and Purpose:** Marrow stromal cells (MSCs) are being tested in clinical trials for stroke patients. MSCs appear to promote recovery through secretomes that promote modulation of immune cells, including myeloid phagocytes. Many stroke patients have comorbidities such as metabolic syndrome, hypertension, hypercholesterolemia, and diabetes for which they are prescribed medications that might affect the function of MSCs and monocytes (Mo) when they are administered in stroke patients. We studied the effects of the two most commonly prescribed stroke medications, statin and statin plus aspirin, on the secretomes of MSCs and their modulation of Mo derived from stroke patients.

**Methods:** Human MSCs, Mo and their co-cultures were exposed to atorvastatin or atorvastatin plus aspirin followed by secretome analysis at 24 h. Monocytes were isolated from healthy controls as well as stroke patients with NIHSS ranging from 11 to 20. Secretome composition was measured using multiplex immunoassay. We used MTT assay to measure proliferation of monocytes. The mixed model was used to analyze experimental data. *p*-values less than 0.05 were considered significant.

**Results:** Atorvastatin and aspirin combination increased the release of IL-1RA from stroke Mo. In MSCs, atorvastatin and aspirin combination reduced the release of pro-inflammatory cytokines such as IL-6, IL-8, MCP-1 and IFN-γ. Atorvastatin alone reduced the release of IL-6, IL-8 and MCP-1 from co-cultures of stroke monocytes and MSCs. Combination of atorvastatin and aspirin had additive effect on reducing the secretion of IL-6 from co-cultures of stroke Mo and MSCs.

**Conclusion:** Atorvastatin, alone and in combination with aspirin can promote anti-inflammatory effect by modulating the secretome profile of Mo and MSCs. Our results suggest that stroke trials involving the use of intravenous MSCs should consider the effect of aspirin and atorvastatin, both of which are administered to the majority of hospitalized ischemic stroke patients.

## Introduction

Stroke is a major cause of death and disability throughout the world. Acute ischemic stroke leads to brisk pro-inflammatory signaling, which eventually causes secondary brain injury. Many types of cell-based therapies (Savitz et al., 2011; Prasad et al., 2014; Moniche et al., 2015; Moniche et al., 2016; Hess et al., 2017) are under development as an investigational treatment for stroke. For example, human bone marrow derived mesenchymal stromal cells (MSCs) may promote recovery after stroke by releasing various biological factors called the secretome which modulate the various immune responses after stroke (Bang et al., 2012; Gu et al., 2016).

Our group has previously studied the effect of aspirin on MSCs as well as in monocytes (Mo) from stroke patients (Satani et al., 2019b). We showed that aspirin has an anti-inflammatory effect on the secretome released from Mo alone, MSCs alone as well as their co-cultures (Satani et al., 2019b). Specifically, it increased the release of Interleukin-1 receptor antagonist (IL-1RA) and decreased the release of IL-6 (Satani et al., 2019b). However, many patients with stroke are also prescribed statin to control cholesterol levels and the effect of statins and combination therapy of statins with aspirin on MSCs, Mo and co-culture has not been documented. As per the American Heart Association and American Stroke Association guidelines, aspirin as well as statins are commonly recommended in patients with ischemic stroke (Kernan et al., 2014). There are many studies which show the positive effects of statins in stroke patients for prevention as well as for improving post-stroke survival (Hebert et al., 1997; O'Regan et al., 2008; Flint et al., 2012). A study in a mouse stroke model showed that withdrawal of a statin led to removal of its protective effects after cerebral ischemia (Gertz et al., 2003). More specifically, simvastatin was shown to augment MSC induced-angiogenesis in a mouse hind limb ischemia model (Zhang et al., 2012).

Patients with an acute ischemic stroke are prescribed medications upon admission to the hospital including aspirin and statin agents. The effects of these drugs in altering long term outcomes after stroke has been well documented; however, the effect of atorvastatin alone as well as atorvastatin and aspirin combination on MSCs is unknown. These interactions are important since clinical trials test intravenous administration of these cells in stroke patients (Lee et al., 2010; Honmou et al., 2011; Kim et al., 2013). Extensive studies have tested MSCs in rodent models of focal ischemic stroke where the timing of administration is 24 h after symptom onset (Satani et al., 2019a). However, clinical trials that would test the IV administration of MSCs in this time frame would involve patients being prescribed both statins and aspirin. After intravenous administration, MSCs interact with various immune cells in the circulation and peripheral organs. Among various peripheral circulating immune cells, monocytes (Mo) play a crucial immunoregulatory role after stroke (Kochanek and Hallenbeck, 1992; Urra et al., 2009). Intravenously injected MSCs could target these Mo directly (Cutler et al., 2010; Melief et al., 2013a). MSCs could help Mo acquire beneficial phenotypes either directly or through its secretome thereby helping in post-stroke repair (Melief et al., 2013b). Hence, we have studied how atorvastatin alone or in combination with aspirin could change secretomes from MSCs as well as Mo, and how these drugs affect the interactions between MSCs and Mo from the blood of stroke patients.

## Methods

### Isolation and Culture of Human Mesenchymal Stromal Cells (MSCs)

MSCs were isolated from commercially available fresh human bone marrow aspirates of a 34 year old male (AllCells, Alameda, CA) using density centrifugation and plastic adherence as previously described (Boregowda et al., 2016). An adherent population of MSCs was obtained 3 weeks after the initiation of cell culture. The cells were screened for typical spindle-like morphology and growth kinetics. These MSCs strongly expressed MSC markers CD73 and CD90, and were negative for hematopoietic markers HLA-DR, CD11b, CD34, CD45, and CD19 as previously described (Kota et al., 2017). The cells were further expanded by plating 1 × 10^6^ passage 2 cells at 200 cells/cm^2^ in 2528 cm^2^ in Nunc^™^ Cell Factory^™^ Systems with complete culture medium (CCM) that consisted of *α*-minimal essential medium (α-MEM; Life Technologies, Grand Island, NY), 17% fetal bovine serum (FBS; Atlanta Biologicals, Norcross, GA), 100 units/ml penicillin (Life Technologies, Carlsbad, CA), 100 μg/ml streptomycin (Life Technologies, Carlsbad, CA), and 2 mM L-glutamine (Life Technologies). At 70% cell confluency, the medium was discarded, the cultures were washed with phosphate-buffered saline (PBS) (Life Technologies, Carlsbad, CA), and the adherent cells harvested with 0.25% trypsin (Life Technologies, Carlsbad, CA) for 5 min at 37°C and frozen at 1 × 10^7^ cells/ml for subsequent experiments as passage 3.

### Collection of Human Blood Samples

Institutional Review Board approved all the studies and protocols involving human subjects. Peripheral blood was collected either from healthy controls or from stroke patients 24 h after presentation of initial symptoms through phlebotomy. Inclusion criteria for stroke patients included any acute ischemic stroke patients with NIHSS between 11 and 20 (Supplementary Table S1).

### Isolation of Human Peripheral Blood Monocytes

Peripheral Blood Mononuclear Cells (PBMCs) of healthy humans and stroke patients were isolated from peripheral human blood by Ficoll gradient. CD14+ monocytes (Mo) were isolated using indirect magnetically labelling technique using a magnetic beads based isolation as previously described (Satani et al., 2019b). Negative selection technique was used where by a cocktail of biotin-conjugated monoclonal antibodies labelled non-monocyte cells such as B-cells, Natural Killer (NK) cells, T-cells, dendritic cells and basophils while non-labelled CD14+ monocytes were collected for further cell culture work.

### Co-Cultures of Mesenchymal Stromal Cells and Monocytes

Isolated monocytes were plated in a 24 well plate at 200,000 cells per well in a serum free DMEM media. 24 h later, they were exposed to different doses of atorvastatin alone (8–80,000 nM), or a combination of atorvastatin, and aspirin (800, 8,000, and 80,000 nM) for atorvastatin and 500, 5000, and 50,000 nM aspirin). Next, MSCs or an equal amount of media were added into each well at 200,000 cells per well to set up a 1:1 contact co-culture. Monocytes exposed to drugs alone without MSCs were used as control. After 24 h of contact co-cultures, media from monocytes exposed to drugs alone or media from contact co-cultures were collected from each well. Collected media was immediately centrifuged at 300 × *g* for 10 min and supernatant was used for secretome analysis. Similar method was used to collect secretomes from MSCs cultured alone in presence of each drug, where MSCs were also plated in 24 well plate at 200,000 cells per well. The volume of media used in each of the 24 wells was 300 µL. Equal volume was used for each conditions.

### Cell Proliferation Assays

MSCs were exposed to various drug concentrations of atorvastatin ranging from 8 nM to 800 µM. At 24 and 48 h of incubation with these drugs, cell proliferation of MSCs was measured using MTT assay by comparing each concentration with the vehicle control.

### Experimental Groups

MSCs were exposed to various drug concentrations of atorvastatin alone or a combination of atorvastatin and aspirin. Atorvastatin alone was tested at concentrations ranging from 8 to 80000 nM. When combination of atorvastatin and aspirin was used, to limit the combinations of drugs, we limited the doses to clinically relevant dose levels. Hence, we studied all possible combinations of atorvastatin and aspirin with following doses: atorvastatin at 800, 8000, 80000 nM, and; Aspirin at 500, 5000, and 50000 nM. In addition, there were following groups: 1) MSCs alone exposed to atorvastatin alone or combination of atorvastatin and aspirin at all doses; 2) Mo alone exposed to atorvastatin alone or combination of atorvastatin and aspirin at all doses; and 3) MSC-Mo co-cultures exposed to atorvastatin alone or combination of atorvastatin and aspirin at all doses.

### ELISA and Multiplex Cytokine Assays

Media from treated MSCs was analyzed for secretomes by using MagPix magnetic bead-based ELISA assay (EMD Millipore). Data were averaged for 3 Mo donors. Briefly, 96-well Magpix plates were used and supernatant media was incubated with magnetic cytokine beads overnight at 4°C. Next day, detection antibodies were added and incubated for 1 h at room temperature. Luminex Magpix plate reader was used to measure the concentrations of IL1-RA, IL-1β, IL-4, IL-6, IL-8, IL-10, Monocyte chemoattractant protein-1 (MCP-1), Tissue necrosis factor alpha (TNF-α), and Vascular endothelial growth factor (VEGF) in the supernatant collected after 24 h of exposure to the drugs. The secretome measured from MSC and Mo was based on our previous work, and we have measured the secretome most relevant to MSC and Mo in this study (Satani et al., 2019b; Satani et al., 2020).

### Statistical Analysis

For the experiments of atorvastatin, we evaluated its dose effect through a mixed model. We applied base-2 logarithm transformation on fold change data to normalize the secretome levels of the interested biomarkers. Mixed models were fitted to the normalized data. In a mixed model, we considered dose level as the fixed effect. Biological replicates on donor were considered as random effect. Based on the mixed model, we estimated log_2_ (fold change) on the secretome levels for different dose levels.

For the experiments involving combination of atorvastatin and aspirin, we derived fold changes on individual secretome levels with respect to vehicle controls of atorvastatin 0 + aspirin 0 nM. Thereby, each individual secretome levels were normalized with appropriate controls. We further applied base-2 logarithm transformation on the fold change data. The transformed data were analyzed by mixed models. The fixed effects of mixed models included atorvastatin concentration, aspirin concentration, and the interaction of these two factors. The donor was treated as random effect mixed models. For accurately estimating the effects of aspirin and atorvastatin in their dose combination groups, we averaged the aspirin concentrations while analyzing the effect of the atorvastatin and vice versa. Based on the mixed models, we estimated the marginal means of log_2_ (fold change) for different concentration levels of atorvastatin and aspirin. All analyses were performed using SAS 9.4 (Cary, NC) and a *p*-value less than 0.05 was considered as significant.

## Results

### Atorvastatin Does Not Alter Proliferation of MSCs at Pharmacologically Relevant Concentrations

We exposed MSCs to doses of atorvastatin ranging from 0.8 nM to 800 µM. We found no significant difference in proliferation of MSCs at any of the atorvastatin concentration as compared to vehicle controls (Supplementary Figure S1). In our previous studies, we found no effect of aspirin alone on MSC proliferation (Satani et al., 2019b).

### Atorvastatin and Aspirin Alter the Release of IL-1RA and TNF-α From Both Healthy Control and Stroke Patients Monocytes

**FIGURE 1:**
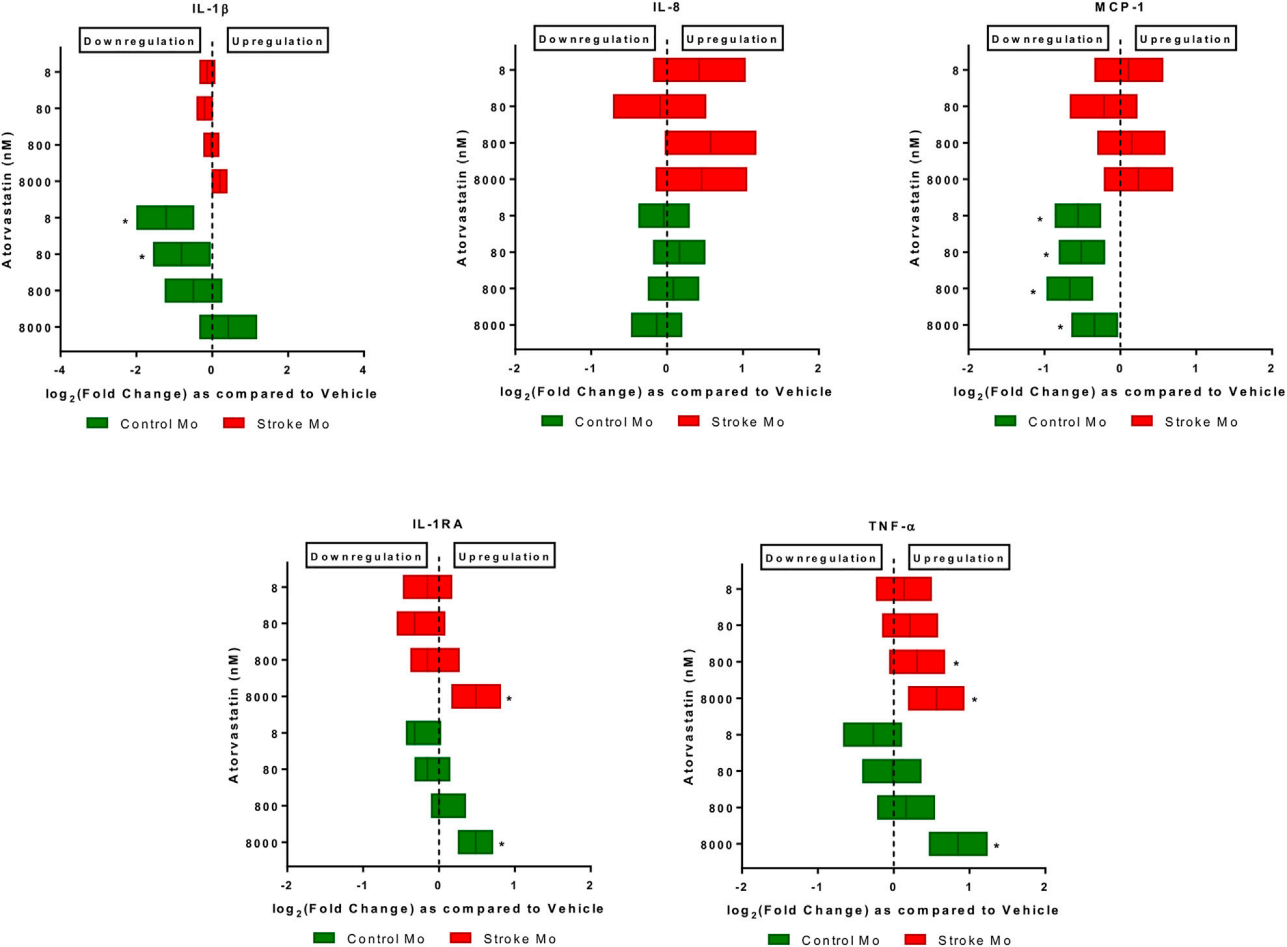
Atorvastatin alters the secretome of monocytes from healthy controls and stroke patients after 24 h of exposure. Atorvastatin increased IL-1RA and TNF-α secretion from stroke patient derived monocytes as well as healthy control monocytes at 8 µM dose. Atorvastatin had no effect on secretions of IL-8, IL-1β and MCP-1 from stroke monocytes, but decreased the MCP-1 secretion from healthy control monocytes. Significance is shown by *p < 0.05.

*Atorvastatin increases the secretion of IL-1RA and TNF-α from stroke monocytes.* In this experiment, we found that atorvastatin in a dose dependent fashion increased the release of IL-1RA and TNF-α from monocytes of stroke patients, as well as healthy control patients (*p* < 0.05, Figure 1). However, the increases of TNF-α release by lower doses of atorvastatin were more robust in case of stroke patient derived monocytes, as compared to healthy donor derived monocytes. Interestingly, atorvastatin reduced the secretion of MCP-1 and IL-1β from healthy monocytes but had no effect on stroke monocytes (Figure 1).

*Atorvastatin plus aspirin increased the secretion of IL-1RA and decreased the secretion of IL-6 from stroke monocytes.* After exposure to different doses of atorvastatin plus aspirin (Table 1), there was a significant increase of IL-1RA release from stroke monocytes (*p* < 0.05). In mixed model analysis testing effects of atorvastatin and aspirin interaction, all three tested concentrations of atorvastatin and all three tested concentrations of aspirin increased the IL-1RA secretions from stroke monocytes, as compared to vehicle control (Table 1). Mixed model also revealed that IL-6 secretion was also decreased from stroke monocytes at 5000 and 50000 nM aspirin with all atorvastatin concentrations (Table 1). Moreover, unlike the increase in TNF-α secretion from Mo upon exposure to highest dose of atorvastatin alone (Figure 1), combination of atorvastatin and aspirin did not affect TNF-α secretion from stroke monocytes, (Table 1).

**TABLE 1:** Estimated fold changes to secretome from stroke patient derived Mo after exposure to combination of atorvastatin and Aspirin with respect to a vehicle control of atorvastatin 0 + Aspirin 0 nM. Combination of atorvastatin and Aspirin increases the IL-1RA secretions from stroke patients Mo at all dose combinations after 24 h of exposure. Significance is shown by *p*-value < 0.05. FC = Fold Change, 95% CI = 95% Confidence Intervals with lower and upper limits.

Stroke patient derived Mo exposed to various dose combinations of atorvastatin and aspirin				
	IL-1β		IL-6	
	FC (95% CI)	*p*-value	FC (95% CI)	*p*-value
Atorvastatin 800 nM + aspirin^†^	0.83 (0.33, 2.08)	0.68	0.43 (0.26, 0.70)	<0.05
Atorvastatin 8000 nM + aspirin^†^	1.15 (0.46, 2.90)	0.76	0.74 (0.46, 1.20)	0.21
Atorvastatin 80000 nM + aspirin^†^	1.26 (0.50, 3.18)	0.61	0.78 (0.48, 1.27)	0.30
Aspirin 500 nM + atorvastatin^§^	1.12 (0.45, 2.83)	0.80	0.89 (0.55, 1.44)	0.61
Aspirin 5000 nM + atorvastatin^§^	0.84 (0.33, 2.11)	0.70	0.50 (0.31, 0.81)	<0.05
Aspirin 50000 nM + atorvastatin^§^	0.92 (0.37, 2.33)	0.86	0.42 (0.26, 0.68)	<0.05

	**IL-8**		**IL-1RA**	
	**FC (95% CI)**	***p*** **-value**	**FC (95% CI)**	***p*** **-value**
Atorvastatin 800 nM + aspirin^†^	0.81 (0.48, 1.38)	0.43	1.46 (1.12, 1.90)	<0.05
Atorvastatin 8000 nM + aspirin^†^	0.71 (0.42, 1.21)	0.20	1.48 (1.14, 1.93)	<0.05
Atorvastatin 80000 nM + aspirin^†^	0.80 (0.47, 1.35)	0.38	1.59 (1.22, 2.07)	<0.05
Aspirin 500 nM + atorvastatin^§^	0.91 (0.54, 1.55)	0.72	1.49 (1.14, 1.95)	<0.05
Aspirin 5000 nM + atorvastatin^§^	0.74 (0.44, 1.26)	0.26	1.58 (1.21, 2.06)	<0.05
Aspirin 50000 nM + atorvastatin^§^	0.81 (0.48, 1.38)	0.42	1.56 (1.20, 2.03)	<0.05

	**MCP-1**		**TNF-α**	
	**FC (95% CI)**	***p*** **-value**	**FC (95% CI)**	***p*** **-value**
Atorvastatin 800 nM + Aspirin^†^	1.03 (0.59, 1.81)	0.92	0.79 (0.39, 1.59)	0.49
Atorvastatin 8000 nM + Aspirin^†^	1.22 (0.70, 2.15)	0.47	1.18 (0.59, 2.40)	0.63
Atorvastatin 80000 nM + Aspirin^†^	1.80 (1.03, 3.15)	<0.05	1.51 (0.75, 3.06)	0.24
Aspirin 500 nM + atorvastatin^§^	1.44 (0.82, 2.52)	0.20	1.25 (0.62, 2.54)	0.52
Aspirin 5000 nM + atorvastatin^§^	1.22 (0.70, 2.14)	0.47	0.91 (0.45, 1.83)	0.77
Aspirin 50000 nM + atorvastatin^§^	1.15 (0.65, 2.01)	0.62	0.88 (0.44, 1.79)	0.72

^†^ Averaged over all aspirin concentrations (0, 500, 5000, and 50000 nM).

^§^ Averaged over all atorvastatin concentrations (0, 800, 8000, and 80000 nM). The averaged effect over all aspirin or atorvastatin concentrations statistically reflects the uniform effect of these drugs in presence of variable doses of the other drug.

### Atorvastatin and Aspirin Alters the Release of Immunomodulatory Molecules From MSCs

**FIGURE 2:**
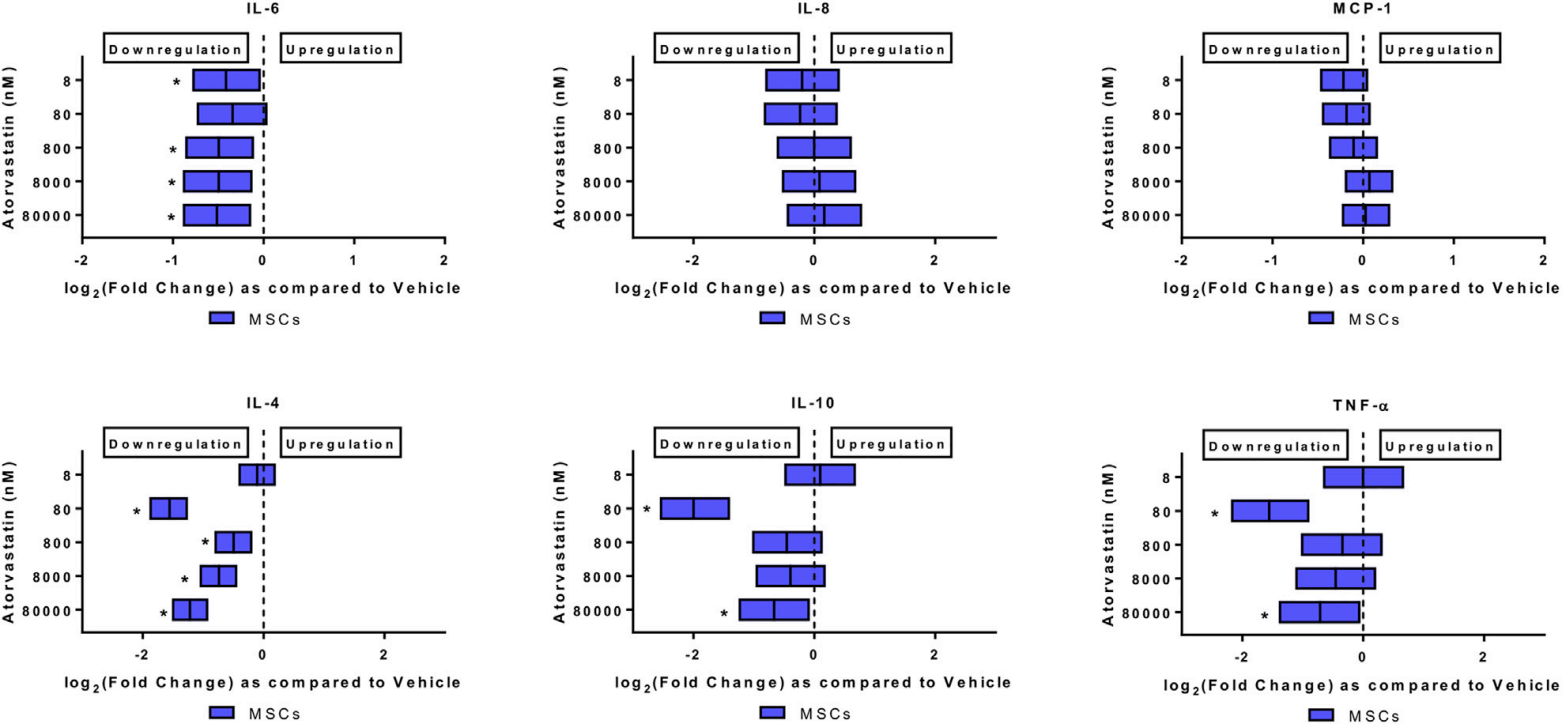
Atorvastatin alters the secretome of MSCs after 24 h of exposure. Atorvastatin decreased IL-6, IL-4, IL-10 and TNF-α secretions from MSCs after 24 h of exposure. Significance is shown by *p < 0.05.

We subjected MSCs to pharmacologically relevant doses of either atorvastatin alone (Figure 2) or a combination of aspirin, and atorvastatin (Table 2). We measured the levels of IL-4, IL-6, IL-8, IL-10, MCP-1, IFN-gamma, and TNF-alpha in secretome.

**TABLE 2:** Estimated fold changes to secretome from MSCs after exposure to combination of atorvastatin and Aspirin with respect to a vehicle control of atorvastatin 0 + Aspirin 0 nM. Combination of atorvastatin and Aspirin decreases the IFN-γ, IL-8, and MCP-1 secretions from MSCs at all dose combinations after 24 h of exposure. In addition, there is also a decrease in IL-6 secretions from MSCs at the highest doses of atorvastatin and aspirin after 24 h of exposure. Significance is shown by *p*-value<0.05. FC = Fold Change, 95% CI = 95% Confidence Intervals with lower and upper limits.

MSCs exposed to various dose combinations of atorvastatin and aspirin				
	IFN-γ		IL-6	
	FC (95% CI)	*p*-value	FC (95% CI)	*p*-value
Atorvastatin 800 nM + aspirin^†^	0.95 (0.84, 0.99)	<0.05	1.07 (0.88, 1.30)	0.52
Atorvastatin 8000 nM + aspirin^†^	0.90 (0.83, 0.98)	<0.05	0.84 (0.69, 1.02)	0.07
Atorvastatin 80000 nM + aspirin^†^	0.73 (0.68, 0.80)	<0.05	0.58 (0.47, 0.70)	<0.05
Aspirin 500 nM + atorvastatin^§^	0.85 (0.79, 0.93)	<0.05	0.83 (0.68, 1.01)	0.06
Aspirin 5000 nM + atorvastatin^§^	0.88 (0.81, 0.96)	<0.05	0.80 (0.66, 0.97)	<0.05
Aspirin 50000 nM + atorvastatin^§^	0.82 (0.75, 0.89)	<0.05	0.82 (0.67, 0.99)	<0.05

	**IL-8**		**IL-1RA**	
	**FC (95% CI)**	***p*** **-value**	**FC (95% CI)**	***p*** **-value**
Atorvastatin 800 nM + aspirin^†^	0.79 (0.69, 0.91)	<0.05	0.72 (0.37, 1.39)	0.34
Atorvastatin 8000 nM + aspirin^†^	0.77 (0.67, 0.89)	<0.05	0.62 (0.32, 1.20)	0.28
Atorvastatin 80000 nM + aspirin^†^	0.65 (0.57, 0.75)	<0.05	0.86 (0.44, 1.66)	0.12
Aspirin 500 nM + atorvastatin^§^	0.86 (0.75, 0.99)	<0.05	0.73 (0.38, 1.42)	0.31
Aspirin 5000 nM + atorvastatin^§^	0.82 (0.71, 0.94)	<0.05	0.70 (0.36, 1.36)	0.15
Aspirin 50000 nM + atorvastatin^§^	0.72 (0.63, 0.83)	<0.05	0.60 (0.31, 1.15)	0.64

	**MCP-1**		**TNF-α**	
	**FC (95% CI)**	***p*** **-value**	**FC (95% CI)**	***p*** **-value**
Atorvastatin 800 nM + aspirin^†^	1.36 (1.09, 1.68)	<0.05	1.04 (0.96, 1.12)	0.36
Atorvastatin 8000 nM + aspirin^†^	0.60 (0.48, 0.75)	<0.05	1.00 (0.92, 1.07)	0.89
Atorvastatin 80000 nM + aspirin^†^	0.32 (0.26, 0.39)	<0.05	0.89 (0.82, 0.96)	<0.05
Aspirin 500 nM + atorvastatin^§^	0.77 (0.62, 0.96)	<0.05	0.97 (0.90, 1.05)	0.41
Aspirin 5000 nM + atorvastatin^§^	0.77 (0.62, 0.96)	<0.05	1.00 (0.93, 1.08)	0.92
Aspirin 50000 nM + atorvastatin^§^	0.79 (0.63, 0.98)	<0.05	0.96 (0.89, 1.04)	0.29

^†^ Averaged over all aspirin concentrations (0, 500, 5000 and 50000 nM).

^§^ Averaged over all atorvastatin concentrations (0, 800, 8000, 80000 nM). The averaged effect over all aspirin or atorvastatin concentrations statistically reflects the uniform effect of these drugs in presence of variable doses of the other drug.

*Atorvastatin alone decreased the levels of IL-6, and TNF-alpha from MSCs.* When MSCs were exposed to atorvastatin, it significantly decreased the secretion of IL-6 and TNF-α (*p* < 0.05, Figure 2). There was also a decrease in IL-4 secretion at all doses and decrease of IL-10 at lower dose (*p* < 0.05, Figure 2).

*Atorvastatin plus aspirin decreased IFN-γ, IL-6, IL-8, and MCP-1 secretions from MSCs.* In mixed model analysis testing effects of atorvastatin and aspirin interaction, there was a significantly reduced secretion of all the pro-inflammatory cytokines including IFN-γ, IL-6, IL-8, and MCP-1 from MSCs at all dose combinations (Table 2). The only notable exceptions were for atorvastatin at 800 nM with all aspirin doses, where MCP-1 secretions increased and for 80,000 nM atorvastatin with all aspirin doses where IL-6 decreased (Table 2).

### Atorvastatin and Aspirin Changes the Immunomodulatory Properties of MSCs-Monocytes Co-culture

**FIGURE 3:**
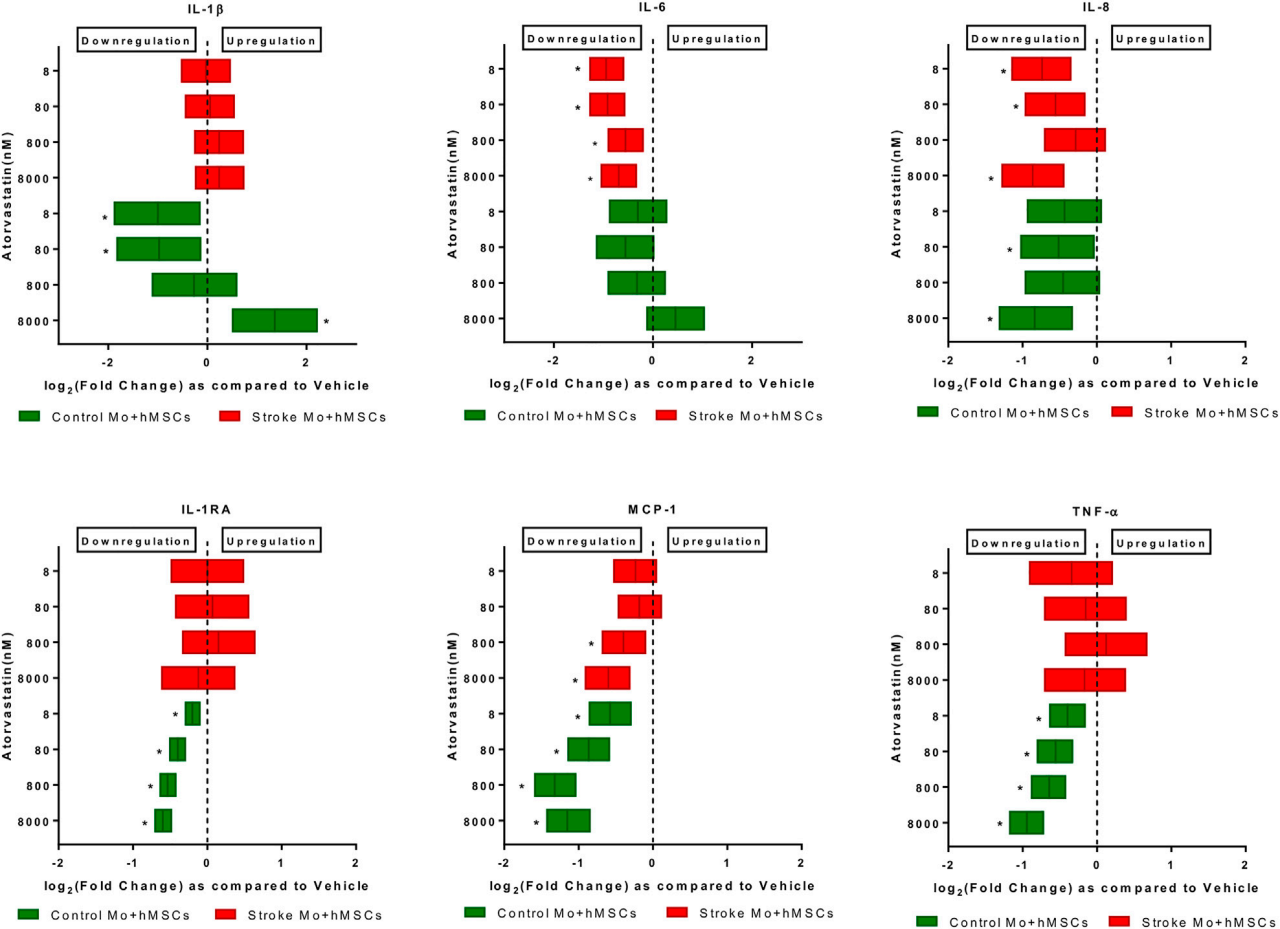
Atorvastatin reduces the cytokine secretions of IL-6, IL-8, and MCP-1 from co-cultures of stroke patient monocytes with MSCs after 24 h of exposure. Atorvastatin also reduces IL-1RA, TNF-α, IL-8, MCP-1 and IL-1β secretions from co-cultures of healthy control monocytes with M-SCs after 24 h of exposure. Significance is shown by *p < 0.05.

It was evident from the above results that aspirin and statins used at pharmacologically relevant concentrations alter secretions of cytokines released from MSCs. To determine whether MSCs exposed to atorvastatin (Figure 3), or atorvastatin and aspirin combination (Table 3) in presence of Mo from control or stroke patients (Figure 3) or stroke patients (Table 3), acquire different immunoregulatory properties, we co-cultured these MSCs with monocytes from stroke patients and analyzed the composition of the secretomes.

**TABLE 3:** Estimated fold changes to secretome from co-cultures of stroke patient derived Mo and MSCs after exposure to combination of atorvastatin and Aspirin with respect to a vehicle control of atorvastatin 0 + Aspirin 0 nM. Combination of atorvastatin and Aspirin decreases the IL-6 secretion from stroke Mo-MSCs co-cultures after 24 h of exposure. In addition, there is also a decrease in MCP-1 secretions at the highest dose of atorvastatin and aspirin after 24 h of exposure. Significance is shown by *p*-value<0.05. FC = Fold Change, 95% CI = 95% Confidence Intervals with lower and upper limits.

Co-cultures of stroke patient derived Mo-MSCs exposed to various dose combinations of atorvastatin and aspirin				
	IL-1β		IL-6	
	FC (95% CI)	*p*-value	FC (95% CI)	*p*-value
Atorvastatin 800 nM + Aspirin^†^	0.86 (0.30, 2.50)	0.78	0.33 (0.09, 1.15)	0.08
Atorvastatin 8000 nM + Aspirin^†^	0.79 (0.27, 2.30)	0.66	0.26 (0.07, 0.92)	<0.05
Atorvastatin 80000 nM + Aspirin^†^	0.81 (0.28, 2.36)	0.69	0.28 (0.08, 0.99)	<0.05
Aspirin 500 nM + atorvastatin^§^	0.78 (0.27, 2.27)	0.64	0.31 (0.09, 1.10)	0.07
Aspirin 5000 nM + atorvastatin^§^	0.98 (0.34, 2.86)	0.98	0.35 (0.10, 1.23)	0.10
Aspirin 50000 nM + atorvastatin^§^	0.45 (0.16, 1.31)	0.14	0.17 (0.05, 0.60)	<0.05

	**IL-8**		**IL-1RA**	
	**FC (95% CI)**	***p*** **-value**	**FC (95% CI)**	***p*** **-value**
Atorvastatin 800 nM + aspirin^†^	1.07 (0.48, 2.38)	0.86	1.33 (0.88, 2.00)	0.17
Atorvastatin 8000 nM + aspirin^†^	0.69 (0.31, 1.52)	0.34	1.33 (0.88, 2.00)	0.17
Atorvastatin 80000 nM + aspirin^†^	0.61 (0.28, 1.36)	0.22	1.44 (0.95, 2.17)	0.08
Aspirin 500 nM + atorvastatin^§^	0.83 (0.38, 1.85)	0.64	1.41 (0.94, 2.13)	0.10
Aspirin 5000 nM + atorvastatin^§^	1.04 (0.47, 2.30)	0.93	1.50 (0.99, 2.26)	0.05
Aspirin 50000 nM + atorvastatin^b^	0.66 (0.30, 1.47)	0.30	1.32 (0.87, 1.99)	0.18

	**MCP-1**		**TNF-α**	
	**FC (95% CI)**	***p*** **-value**	**FC (95% CI)**	***p*** **-value**
Atorvastatin 800 nM + aspirin^†^	0.98 (0.43, 2.22)	0.95	1.13 (0.57, 2.24)	0.72
Atorvastatin 8000 nM + aspirin^†^	0.55 (0.24, 1.24)	0.14	0.94 (0.47, 1.86)	0.85
Atorvastatin 80000 nM + aspirin^†^	0.18 (0.08, 0.42)	<0.05	0.72 (0.36, 1.42)	0.33
Aspirin 500 nM + atorvastatin^§^	0.55 (0.24, 1.25)	0.15	1.06 (0.53, 2.10)	0.87
Aspirin 5000 nM + atorvastatin^§^	0.71 (0.31, 1.63)	0.41	0.97 (0.49, 1.93)	0.94
Aspirin 50000 nM + atorvastatin^§^	0.49 (0.22, 1.12)	0.09	0.70 (0.35, 1.39)	0.30

^†^ Averaged over all aspirin concentrations (0, 500, 5000 and 50000 nM).

^§^ Averaged over all atorvastatin concentrations (0, 800, 8000, 80000 nM). The averaged effect over all aspirin or atorvastatin concentrations statistically reflects the uniform effect of these drugs in presence of variable doses of the other drug.

*Atorvastatin reduced the IL-6, IL-8, and MCP-1 secretion in co-cultures of MSCs and human monocytes.* When monocytes from stroke patients were co-cultured with MSCs in presence of atorvastatin (8–8000 nM), we found that IL-6, MCP-1, and IL-8 secretion was reduced at almost all atorvastatin doses (*p* < 0.05, Figure 3). In case of co-culture of MSC plus Mo from control monocytes, atorvastatin reduced secretion of IL-1b (only at lower doses), IL-8, IL-1RA, MCP-1, and TNF-α (across the doses).

*Atorvastatin plus aspirin significantly decreased IL-6 secretions from co-culture of stroke Mo and MSCs.* After exposure to both atorvastatin plus aspirin at different doses, there was a significantly reduced secretion of IL-6 from co-cultures of Mo and MSCs. In mixed model analysis testing effects of atorvastatin and aspirin interaction, higher doses of atorvastatin (8000 and 80000 nM) and aspirin (50000 nM) decreased the release of IL-6 from these co-cultures as compared to vehicle control (Table 3). There was up to 2-fold decrease in IL-6 secretion from these co-cultures.

## Discussion

Trophic factors including growth factors and cytokines have an important role underlying the potential benefits of MSCs as cell-based therapy for ischemic stroke (Drago et al., 2013; Teixeira et al., 2013; Satani and Savitz, 2016). The number of passages, storage conditions, and types of solvents of MSCs impact the viability and immunomodulatory effects of MSCs (François et al., 2012; Gruber et al., 2012; Nikolaev et al., 2012; Lee et al., 2014; Yang et al., 2016; Parsha et al., 2017), however effects of medications on MSCs have not been studied in much detail. MSCs have been actively tested in clinical trials as well (Lee et al., 2010; Honmou et al., 2011; Kim et al., 2013). Leaders in the field of stroke from both academia and industry have identified the need to study effect of these medications on MSCs and recently recommended studies in their STEP4 guidelines (Boltze et al., 2019). Since intravenously administered MSCs have advanced to clinical trials in stroke patients, we posed clinically relevant questions about the effects of aspirin and atorvastatin, the two most commonly prescribed medications to patients hospitalized for ischemic stroke. We sought to evaluate the effects of these medications on MSCs by specifically studying MSC derived secretomes and the immunomodulatory effects of MSCs on monocytes. A range of drug concentrations were studied to simulate clinically relevant drug ranges in a patient’s bloodstream and to assess for dose-dependent effects. In addition, IL-1β, IL-4, IL-6, IL-8, MCP-1, IL-1RA, and TNF-α were measured. The normal plasma levels of these cytokines are shown in Supplementary Table S2 (Jones et al., 1997; Kleiner et al., 2013; O'Neill et al., 2013; Li et al., 2018).

Aspirin reduces the risk of stroke by about 15% at doses ranging from 50–1500 mg per day (Johnson et al., 1999). The therapeutic dose for aspirin is up to 300 ug/mL. Doses greater than that producing plasma levels of 700 and 1400 µg/mL are considered toxic (Arif and Aggarwal, 2019). Therapeutic plasma levels of aspirin can range from 3 to 10 mg/dl. These doses translates to doses less than 50 µM. Hence we chose 50, 5µM, and 500 nM doses for our experiments. Atorvastatin is an inhibitor of enzyme 3-hydroxy-3-methylglutraryl-coenzymeA (HMG-CoA) reductase. Atorvastatin is an FDA approved drug to prevent cardiovascular events in patients with cardiac risk factors and is commonly given to stroke patients (McIver and Siddique, 2020). It is commonly given to patients with the dose between 10 and 80 mg per day. The plasma levels of atorvastatin ranges from 20–200 ng/ml (Lins et al., 2003). With multiple dosing these levels can increase to higher than 4 µg/ml which translates to approximately 8000 nM dose. Since plasma levels of atorvastatin varies based on dosing, to cover all ranges of plasma concentrations, we studied 800, 8,000, and 80,000 nM doses of atorvastatin.

We have previously shown that aspirin significantly increases release of IL-1RA from monocytes (Satani et al., 2019b). In our current study, we also noted that atorvastatin increased the secretion of IL-1RA from stroke patient derived monocytes at the concentration higher than 8000 nM. Based on this findings, we wanted to study if a combination treatment of atorvastatin and aspirin poses a differential effect on IL-1RA secretion. In mixed model analysis testing effects of atorvastatin, aspirin and its interaction, all three tested concentrations of atorvastatin and all three tested concentrations of aspirin increased the IL-1RA secretions significantly from stroke monocytes as compared to vehicle control. IL-1RA secretion was increased by about 50% at all dose combinations of atorvastatin with aspirin, suggesting a possible additive effect of both drugs on stroke monocytes. IL-1RA is a member of interleukin 1 cytokine family, and is known to block IL-1 receptor and inhibit pro-inflammatory effect of IL-1β. Increased IL-1RA has been shown to exert a neuroprotective effect in experimental stroke models (Clausen et al., 2016). Increased IL-1RA has been associated with reduced infarct size and improved functional outcome in stroke (Clausen et al., 2016). Subcutaneously administered IL-1RA, in a randomized controlled phase 2 trial, showed that IL-1RA reduced plasma inflammatory markers which are known to be associated with worse clinical outcome in ischemic stroke. Subcutaneous IL-1RA was safe and tolerated well (Smith et al., 2018). It is already well established that MSCs can skew monocytes toward anti-inflammatory pathways (Melief et al., 2013b). Our previous studies have already showed that MSCs can change the secretome to make it anti-inflammatory and pro-regenerative in presence of other clinical drugs (Satani et al., 2017; Satani et al., 2018; Satani et al., 2019b; Satani et al., 2020). These anti-inflammatory monocytes have been known to induce regulatory T cells, which further provide benefits after ischemic stroke (Melief et al., 2013b). Since our study found that a combination of atorvastatin and aspirin specifically increases IL-1RA in stroke monocytes, this could point to a potential beneficial effect of giving atorvastatin and aspirin to stroke patients.

Our previous study also showed that aspirin increased secretion of anti-inflammatory cytokine IL-4, and reduced secretion of pro-inflammatory cytokine IL-6 from MSCs (Satani et al., 2019b). In addition, MCP-1 was also significantly increased from MSCs upon exposure to aspirin (Satani et al., 2019b). These results suggest that aspirin alters the secretomes of MSC in favor of increased anti-inflammatory factors. After aspirin, statins are the second most commonly prescribed medication administered to hospitalized stroke patients to reduce LDL and to prevent secondary stroke (Castilla-Guerra et al., 2016). When MSCs were exposed to atorvastatin at doses ranging from 8 to 8000 nM, atorvastatin showed a two-fold anti-inflammatory effect by reducing the secretions of inflammatory cytokines such as IL-6, and TNF-α. Consistent with these results, we also found a significant reduction in IFN-γ, IL-6, IL-8, and MCP-1, when both aspirin and atorvastatin were given. In our mixed effect model, all clinically relevant doses of atorvastatin given within clinically relevant ranges of aspirin showed significantly reduced secretion of pro-inflammatory cytokines. In addition, in our mixed effect model, we also found that the combination of atorvastatin and aspirin decreased IL-6 secretion from co-cultures of stroke Mo and MSCs. Statins have been shown to have reparative effects even without MSCs (Park et al., 2016). A systematic review by Park et al. in 2016 found statins improved cell-based repair of organ injury (Park et al., 2016). In addition, preclinical studies have shown the benefits of combining statins with MSCs. Cui et al. showed that combining MSC treatment with simvastatin in a rat model of ischemic stroke significantly improved neurological outcome and enhanced angiogenesis as well as arteriogenesis (Cui et al., 2009). Combination therapy of statins and MSCs also improved neurological function in rats after traumatic brain injury when statins were administered at an optimal dose (Mahmood et al., 2008). Our results raise the possibility that the combination of statins and MSCs increases the immunomodulatory effects of MSCs and may explain why statins have been more effective than MSCs alone to reduce neurological deficits in animal models of stroke. In addition, when we combined our previous results with aspirin (Satani et al., 2019b), it raised a possibility that combination therapy with aspirin and atorvastatin might be more pro-regenerative. There are several limitations in our study. First, the MSCs used in our study come from a single donor. However, the intention of this study was to evaluate the changes in immunomodulatory properties of MSC and/or Mo after exposure to a combination of aspirin and atorvastatin. Since aspirin and atorvastatin are the two most commonly prescribed drugs in stroke patients, our study will provide useful information for designing future clinical trials. Although MSC donor variability is described in literature in detail, adding MSC donor variability to this study design would have gone beyond the scope of this work, which adequately addresses the hypothesis that a combination of atorvastatin and aspirin enhances the pro-regenerative interactions of MSC and Mo.

Taken together, our results show that combination treatment with atorvastatin and aspirin at clinically relevant doses have significant effects on the secretomes and immunomodulatory signaling of MSC. Since immunomodulation is an important mechanism of how MSCs promote stroke recovery in animal studies, our results suggest that atorvastatin and aspirin combination may exert drug interactions on MSCs and peripheral Mo. Exposure to these medications may be an important variable that should be considered in planned clinical trials testing MSCs in stroke patients.

## Summary/Conclusion

Exposure of MSCs to clinically relevant drugs can alter their immunomodulatory function. Our results suggest that stroke trials involving use of intravenous MSCs should consider the impact of atorvastatin and aspirin in modulating the secretome. Our results indicate a possibility that patients on atorvastatin and aspirin may respond better to MSC treatment as compared to those patients not on these medications.

## Data Availability

The raw data supporting the conclusions of this article will be made available by the authors, without undue reservation.
