# Supplementary material for: A Combination of Atorvastatin and Aspirin Enhances the Pro-Regenerative Interactions of Marrow Stromal Cells and Stroke-Derived Monocytes In Vitro
Source: Front Pharmacol. 2021 Apr 20;12:589418. doi: 10.3389/fphar.2021.589418 (PMC8093790; doi:10.3389/fphar.2021.589418)
Supplement: Supplementary file 1 [file datasheet1.pdf]

Supplementary Material

A Combination of Atorvastatin and Aspirin enhances the pro-regenerative interactions of marrow stromal cells and stroke-derived monocytes *in vitro*

Nikunj Satani[1#], Xu Zhang[2], Kaavya Giridhar[1], Natalia Wewior[1], Chunyan Cai[2], Jaroslaw Aronowski[1], Sean I. Savitz[1]

[1]Institute for Stroke and Cerebrovascular Diseases, McGovern Medical School at UTHealth, Houston, Texas, USA
[2]Center for Clinical and Translational Sciences, McGovern Medical School at UTHealth, Houston, Texas, USA

[#]Corresponding Author:
Nikunj Satani, MD, MPH
Institute of Stroke and Cerebrovascular Diseases
McGovern Medical School at UTHealth
6431 Fannin Street, MSB 7.628, Houston TX 77030
Phone: 713-500-5512
Email: Nikunj.B.Satani@uth.tmc.edu

Figure S1. Clinically relevant concentrations of Atorvastatin does not alter cell proliferation of MSCs at physiological concentrations. MTT assay was performed after 24 and 48 hours of exposure to Atorvastatin. Absorbance was measured at 595nm. Data is normalized with vehicle control (Dose 0).

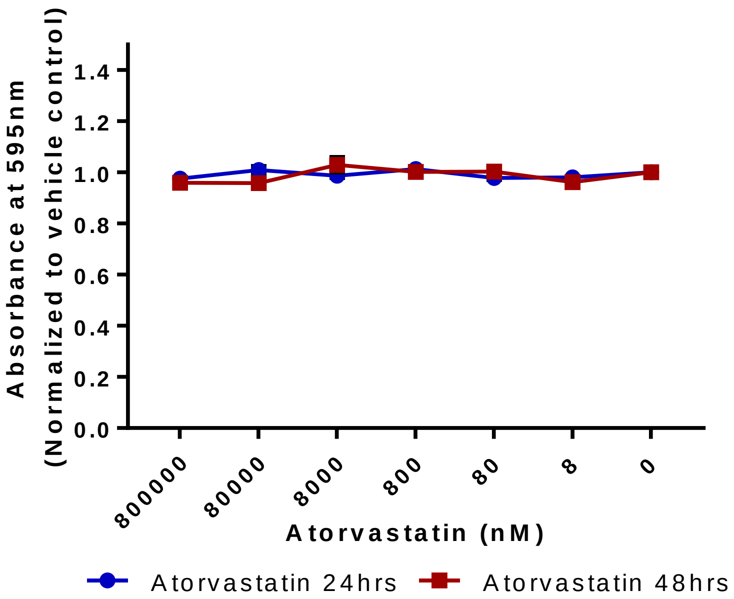

Figure S2. Atorvastatin does not change the cytokine secretions of IL-4 and VEGF from co-cultures of stroke patient monocytes with MSCs after 24 hours of exposure.

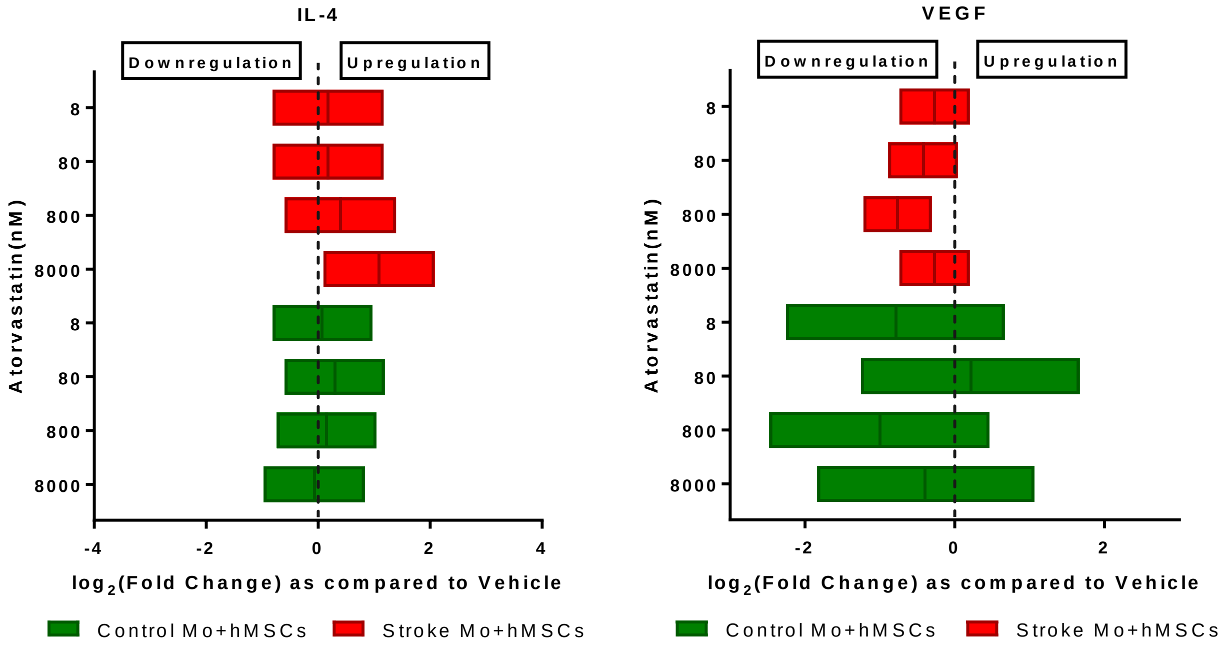

Table S1. National Institute of Health Stroke Scale (NIHSS) for the stroke patients from where the peripheral blood monocytes were collected.

Enrolled Stroke Patient from where peripheral blood monocytes were isolated	Diagnosis at discharge	NIHSS at admission
Stroke patient #1 (Monocytes #1)	Acute Ischemic Stroke	11
Stroke patient #2 (Monocytes #2)	Acute Ischemic Stroke	12
Stroke patient #3 (Monocytes #3)	Acute Ischemic Stroke	15

Table S2. Normal plasma levels in a healthy subject for all cytokines measured. *ND = not detectable*

Cytokine	Normal levels (pg/ml)
IL-1β	0.5 - 12.0
IL-4	ND - 1.5
IL-6	0.5 - 5.0
IL-8	24.4 - 35.9
MCP-1	20.1 - 78.9
IL-1RA	101.2 - 172.0
TNF-α	ND - 8.1
